# From eco-anxiety to eco-hope: surviving the climate change threat

**DOI:** 10.3389/fpsyt.2024.1429571

**Published:** 2024-10-18

**Authors:** Sophia Betro’

**Affiliations:** Istituto di Psicopatologia, Rome, Italy

**Keywords:** climate change, mental health, eco-anxiety, eco-hope, ecological degradation, interdisciplinary

## Abstract

**Introduction:**

As the average global temperature increases, the effects of climate change worsen, through effects on worsening extreme events as well as exacerbating political, economic, and social turmoil (wars, conflicts, and migrations). This poses an existential risk to the survival of humans and non-humans. These effects are visible due to the impact on people’s mental health and psychophysical well-being. This article aims to explore the growing phenomenon of psychoterratic syndromes, with focus on the effect of eco-anxiety on mental health. Furthermore, the relationship between eco-anxiety and behavior response (both individual and collective) in the climate crisis era is outlined.

**Methods:**

A research with interdisciplinary approach was carried out for recent literature and articles relating to psychoterratic syndromes and the effects of climate change on mental health.

**Results:**

The article explores the effects of climate change on mental health, including various research on the onset of new emotions in response to psychological effects to climate change, called psychoterratic syndromes (such as eco-anxiety, climate anxiety, solastalgia, eco-grief). Among these, eco-anxiety is the most popular term used for describing how people feel about climate change. However, the paradigm that described eco-anxiety only as a pathological emotion needs to be changed.

**Discussion:**

The article emphasizes the positive effect of eco-emotions and the need to stimulate people to move from a state of anxiety, which could bring apathy and resignation, toward eco-hope. Eco-hope could be an adaptive coping mechanism in people and communities, which is key to preventing, mitigating, and protecting mental and planetary health.

## Introduction to climate change

We are facing moments of great difficulty that are occurring rapidly in which each month is the hottest ever recorded, or each year is the warmest on record temperatures (1). There is more evidence that climate change is human-caused and is affecting weather and climate globally by raising in global temperature (2). These effects occur upon an increase in intensity and frequency of phenomena such as heatwaves, heavy precipitation, droughts, and tropical cyclones. By rise in temperature, these phenomena create an “*existential threat to the planet*” (3), to the point that *“the era of global boiling has arrived*” (4). The 1.5 Celsius degree limit set by the Paris Agreement in 2015 is a necessary set because beyond this threshold, the existential threat becomes real for many people, like those living in the Marshall Islands of the Pacific (5). Their present and future are threatened by the rising sea level, placing them in great vulnerability as the ocean is as much a part of their landscape as the land itself. The National Adaptation Plan is a survival plan designed to address this threat by reducing vulnerability and integrating adaptation strategies to cope with climate change (6).

There arises the need to planning our survival and preparing ourselves for future climate events, recognizing the necessity of both preventing and adapting to such events and climate change. What is happening in the Marshall Islands can be viewed as an alarm that eventually all of us will face.

We are witnessing a transformation of our system where there is an emerging need to broaden our frameworks to accommodate the variations and new scenarios arising from climate change.

The increasing temperature fluctuations have required an adjustment in our reference system. For example, the color purple has been incorporated into the warning stripes, which are data visualization graphics utilizing a series of colored stripes arranged chronologically to visually represent long-term temperature trends. This addition complements the dichotomous blue and red colors used to denote temperature variations visually (7). Furthermore, there has been a discussion about the necessity of introducing a category 6 to the classification of cyclone intensity and speed (8).

In a complex system like the planet, where every human and non-human element is interconnected, projections are made to anticipate the future. The climate and biosphere form a non-linear system in which chain reactions and a domino effect that undermine planetary balance can easily occur. With the raise of temperature beyond 1.5°C, we are rapidly approaching tipping points (9).

The anthropogenic perturbations of the global environment are often addressed as separate issues, such as climate change, biodiversity loss, or pollution. However, this approach overlooks the non-linear interactions between these perturbations and their aggregate effects on the overall state of the Earth system. Instead, we must consider the state of the Earth system as a whole (10). We need to address the events as “compound hazards,” where it is essential to analyze the interactions between climate hazards and drivers because various aspects in the real world influence each other and intersect. For example, the destruction of electrical infrastructure by a cyclone can lead to unsanitary conditions or disruptions in healthcare services, increasing the risk of disease outbreaks or difficulties in managing a heatwave (11). A recently experienced compound hazards involve the connection between climate change and the COVID-19 pandemic (12). In the compound hazard, we must include the effect on mental health in people and communities.

The catastrophic impacts of climate damages have effects on the breakdown of communities or societies, disrupting socioeconomic and political conditions, exacerbating social turmoil such as wars, conflicts, and forced migrations (13). The inevitable impact extends to mental health because we cannot consider the Earth’s systems separate from its inhabitants.

## Effect on mental health—what we know

People could experience psychological distress by facing the uncertainty, unpredictability, and uncontrollability of climate change and ecological degradation (14).

There has been extensive research conducted on the climate change in recent years (2). The extreme events are classified from acute to chronic. Acute events are defined as rapid-onset disasters, such as extreme weather events (typhoons, cyclones, floods, wildfires, heatwaves), whereas subacute events include slow-onset events such as droughts. Slow environmental changes manifest as chronic events, such as rising sea levels and the potential future disappearance of islands (Pacific Islands) or cities, loss of biodiversity, or mass extinction. All these events entail both direct and indirect impacts that can coexist, overlap, and be interconnected, especially for their impact on mental health and social system (15).

The impact of climate events on individuals can also be divided into direct or indirect effects (16). Direct effects occur when individuals have experienced a climate-related extreme event such as a flood or tornado. The indirect effects on mental health due to climate change include witnessing calamities, living near affected areas, or learning about an event, even from a distance (17). The results on the well-being and psychophysical health of individuals ranging from distress to clinical disorders such as depression, anxiety, posttraumatic stress disorder (PTSD), insomnia, and suicidal thoughts (18).

For instance, heat stress caused by heatwaves has been associated with mood disorders, anxiety (19), rising of suicide rates (20), and aggressive disorder (21). People with mental illness have a greater risk to death or hospitalization during heatwaves (22).

Furthermore, people who lived and experienced floods or tornado and hurricanes have consequences on mental health as well. It has been seen that survivors of these events have a greater risk of developing PTSD, depression, and anxiety (23, 24), as a result of mourning, displacement, lack of access to medical care (25), and psychosocial stress due to loss of lives and possessions (26, 27).

It is also known that there is variability depending on the type of climatic event and other determinants such as vulnerability to extreme events, where those who are most vulnerable are subject to the worst mental health consequences (such as women, children, elderly people, and people with preexisting health problems or psychiatric disorders) (17).

## Effect on mental health—new category

The search on the PubMed/MEDLINE/ResearchGate/Google scholar database was conducted starting from the combination of the following keywords: “climate change,” “climate crisis,” “mental health,” “health,” “eco-emotion,” “climate emotions,” “psychoterratic syndrome,” “ecoanxiety,” “hope,” “ecohope,” “scoping review,” and “review”. The search included all languages, and we focus on articles written in English. The resulting titles and abstracts were screened selecting publications centered on the topic of the eco-emotion’s consequences on mental health. The exclusion criteria are as follows: Language (non-English articles excluded); No explicit focus on place; No substantive discussion of, or reference to, mental health/emotion/climate change; No explicit empirical, theoretical, or practical focus on place.

Alongside the direct effect on mental health from extreme events, climate change also leads to landscape modification, through global temperature increases, droughts, and changes in the atmosphere and the environment. This has an impact on people’s mental health and well-being.

The discussion about the need to introduce new terminology to describe this mental shift has emerged in recent years. These terms are referred to as “eco-emotions” or “climate emotions” but also as “psychoterratic syndromes,” as defined by Albrecht (28). There is an etymological difference between eco-emotions and climate emotions (see for more details (29)), but I will always refer to eco-emotions in the text for simplicity.

The psychoterratic syndromes are defined as Earth-related mental syndromes where people’s mental well-being (Psyche) is threatened by the severing of healthy links between themselves and their home/territory (Terra).

The first concept of psychoterratic syndromes was the term “solastalgia” introduced by Glenn Albrecht in 2003. From this date, a growing interest in solastalgia began, with a large number of articles published (30). From this concept, many others have been added in response to the emotions that arise when we confront the effects of climate change (31). Over the years, Albrecht has included various eco-emotions related to psychoterratic syndromes such as solastalgia, eco-anxiety, climate anxiety, eco-trauma, and eco-grief, ecological stress, and eco-worry (32).

These definitions have expanded over the years to describe the various mental states resulting from the impact of climate change (29). Feelings and emotions in response to climate change may range from healthy and constructive psychological responses to chronic fear about cataclysmic environmental events (33). Eco-anxiety is the most commonly used term, both in the population and in the various studies and articles published on the effects of climate change. I will therefore only refer to eco-anxiety in the article. For Albrecht, the term “eco-anxiety” refers to a chronic fear of environmental doom (28). Eco-anxiety is also defined as mental distress or anxiety associated with worsening environmental conditions or anxiety experienced in response to the ecological crisis (34). Eco-anxiety has become an umbrella term used to describe various emotions such as fear, anger, exhaustion, powerlessness, feelings of loss, helplessness, and phobia and despair (35).

Generally speaking, emotions can be seen as a continuum ranging from worry to anxiety, to fear and anger or trauma, intensifying toward clinically significant responses that require specific support or result in emotional, cognitive, social, or functional impairments (36). Like any form of anxiety, when eco-anxiety is characterized by severe and debilitating worrying, it can be maladaptive and potentially lead to the development of anxiety disorders (37). Because of the complexity and severity of ecological problems, eco-emotions, especially eco-anxiety, are often perceived as maladaptive and paralyzing forms that lead to climate apathy, depression, and an inability to take action (38). Eco-anxiety is currently not considered a mental health disorder and has no formal guidelines for clinical diagnosis or treatment (39).

The constant exposure to news about climate events, with dates and deadlines “by 2030” or “by 2050” and actions to limit carbon emissions and the temperature “below 1.5 Celsius degree” or “below 2 Celsius degrees,” could lead population to a sense of despair, hopelessness, and inaction (40).

Instead of promoting change and encouraging action, a climate fatalism is encouraged. It means a personal belief that climate change is unstoppable and changing the way of doing does not matter. This lack of efficacy encompasses self-efficacy (the belief in an individual’s capability to achieve a task) or response efficacy (the belief that a behavior will yield the intended outcome) (41). People can also develop denial, as a defense mechanism to ward off overwhelming emotions (42). It is a counterproductive effect by presenting climate change in such an alarmist manner.

Here, we are experiencing and feeling the effects of climate change in our psyche, and hence, there is a need to broaden our narrative to describe what is happening within us. This effect impacts people’s mental health, and there is an increasing need and demand to clarify effects on people’s emotions.

## Moving forward emotions—communicate the eco-emotions

Emotions are a crucial component of a cognitive feedback system that steers responses to challenging decision-making problems, and anxiety or eco-anxiety is no exception to this (43).

In fact, eco-anxiety could be seen as an adaptive response to the future-oriented threat of climate change. Engaging with this emotion is, for example, associated with pro-environmental behavior (37). In this way, changing perspective, eco-anxiety emerges as “practical anxiety”: It prompts people to consider what would be the best course of action. It can be a driving force for future decisions that benefit the planet in combating climate change (44). Instead of being overwhelmed by what cannot be done or changed, people could engage in pro-environmental behavior, in actions taken by individuals or groups that aim to minimize harm to the environment and promote sustainability other than well-being. Furthermore, while feelings of helplessness may discourage individuals from addressing health or other individual-level risks, fostering hopefulness can serve as a catalyst for proactive engagement (45). Here is how transitioning from eco-anxiety and engaging in pro-environmental behaviors to cultivating eco-hope as eco-emotions can be a key to greater personal and community resilience.

We have to find a balance between climate fatalism and the overly strong emphasis on optimism. Hope is a key element for survival and emotional resilience during climate change (46).

Hope is not a unique and univocal concept. It encompasses different natures, like cognitive or emotional (47), and values that are subjective (48). Measuring hope through scales (quantitative study) or questionnaires (qualitative study) has often been seen to correlate with action with respect to climate change. Hope is different from optimism, which can lead to a reduction of action by thinking that everything will eventually work out (47). It is not possible to rely on an optimism at the risk of minimizing the reality of the current situation.

One standard definition of hope is “*the perceived capability to derive pathways to desired goals, and motivate oneself via agency thinking to use those pathways*” (49). Hope requires a belief that the object of our desire is possible but uncertain, like what we experience with the word future in face on climate change. Having hope is to have a direction toward the future with a different predisposition compared with anxiety (48).

It is possible to cultivate eco-hope through various interventions to reduce eco-anxiety and increase personal resilience in the face of climate events. These interventions act on several personal factors. For example, the therapist could increase the client’s internal resilience through various cognitive interventions (35). Through interventions done in groups, people can experience greater social connection and emotional support. This encourages people to more active interventions that can lead to a greater connection with nature (35).

Engaging in any form of pro-environmental behavior increases an individual’s self-efficacy, which is the perception of their ability to effect positive change regarding the environment and to be in control of whether they would act to prevent harmful outcomes (50).

It is important that therapist or media communication promotes and informs about the importance of engaging in pro-environmental actions, whether in private or public forms. This action can serve as a catalyst for climate activism and promote emotional well-being.

For coping strategies, it is important to shift from problem-focused coping to meaning-focused coping. Problem-focused coping involves encouraging individuals to take action and seek social connections and emotional support by joining groups (51), as seen before. However, doing so can be difficult in environments with many stressors and variables that are difficult to control, such as climate change. Instead, by promoting meaning-focused coping, individuals could rely on their beliefs, values, and existential goals to sustain coping and well-being during times of chronic stress. This strategy can be particularly advantageous when facing the challenges of climate change (51). Aligned with the notion that feeling hopeful about taking action is crucial for actually engaging in action, many findings suggested the significance of experiencing hope when considering engaging in climate action.

Hope is strengthened when individuals consistently achieve their goals, but it diminishes when their efforts to reach those goals are repeatedly hindered. People who cultivate hope feel better than people who do not, and are more inclined to action (52). Hope not only drives action, but action in turn fosters hope (47).

Hope interacts with other emotions like worry or fear of the future in helping the activist to continue facing the climate-problem and negative emotions in an active way. Eco-hope is also a complex emotion with differences in meaning that vary from gender to ideological, cultural, or personal differences and so on, which could moderate the influence from hope interventions (47).

There is more and more news about a nascent activism, especially among young people (52). Indeed, young people are more prone to the effect of climate change, both in feeling of eco-anxiety and in the consequences of extreme events (53).

## Conclusion

As we saw, there is extensive evidence of eco-hope’s pivotal role in safeguarding mental well-being and nurturing activism especially in climate crises. In the context of governmental and political inaction, climate hope symbolizes resilience, strength, and a will to change (40), enabling psychological and emotional responses to the climate emergency to be transformed into action (54).

Hope presents a clear divergence from denial, avoidance, or apathy. Active hope demands that we confront two opposing truths: the gravity of the crises and the promise of change and a sustainable future through prompt collective action. Embracing this realistic hope alongside the distress of eco-anxiety can fortify resilience in people (55).

Hope motivates individuals to devise strategies to achieve their goals, making it a valuable therapeutic tool in clinical settings (40). Balancing truth and hope in eco-emotions necessitates active engagement with action, as highlighted by the concept of “active” or “constructive” hope (56).

We can envision different directions for our future. In one scenario, eco-anxiety and eco-emotions are not listened to but rather stigmatized. We ignore the signals that our bodies are trying to send us, like the signals of our planet that are sending in these years, and we do not know how to react. If we think of the Earth as a large organism, then the signals coming from various sources and the alarms we see, if ignored, lead to a worsening situation.

On the other hand, we can use and harness our emotions as a driving force to act, to promote pro-environmental behaviors and climate hope, for resilience and adaptation to climate change. Using hope to enhance personal and planetary well-being involves embracing a positive vision of the future, fueling motivation and commitment to enact meaningful change. Individuals and communities can cultivate hope through concrete actions aimed at preserving the environment, promoting sustainability, and addressing environmental threats, but also designing the future, as well as predicting and formulating adaptation plans for probable environmental disasters. Cultivating people’s hope and resilience is the first step toward better mental well-being.

Future research should be focused not only on eco-anxiety but also on eco-hope in people and the underlying mechanisms. It is crucial to understand how to communicate and promote this type of emotion as mental health practitioners, for the well-being of people and planet.

Research is needed for a clear definition of eco-hope related to climate change. Also, studies are necessary for qualitative and quantitative methods for a major understanding of this conceptual construct of eco-hope. Also, there is the need to investigate the differences between genders, between populations in some regions of the world (such as the global south), and specific population groups (such as youth or Indigenous peoples) that could moderate the influence from hope interventions.

As mentioned in the Introduction, it was necessary to introduce new categories to describe the global effects of climate change. Therefore, it is necessary to do regarding mental health, by introducing new categories and new theorizations that help us in coping with the effects of climate change.
